# AFF4 regulates cellular adipogenic differentiation via targeting autophagy

**DOI:** 10.1371/journal.pgen.1010425

**Published:** 2022-09-23

**Authors:** Yaqian Chen, Qiwen Li, Yuting Liu, Xuelan Chen, Shuang Jiang, Weimin Lin, Yuning Zhang, Rui Liu, Bin Shao, Chong Chen, Quan Yuan, Chenchen Zhou

**Affiliations:** 1 State Key Laboratory of Oral Diseases, National Clinical Research Center for Oral Diseases, West China Hospital of Stomatology, Sichuan University, Chengdu, China; 2 Department of Hematology, State Key Laboratory of Biotherapy and Cancer Center, West China Hospital, Sichuan University, Chengdu, Sichuan, China; University of Michigan, UNITED STATES

## Abstract

Transcriptional elongation is a universal and critical step during gene expression. The super elongation complex (SEC) regulates the rapid transcriptional induction by mobilizing paused RNA polymerase II (Pol II). Dysregulation of SEC is closely associated with human diseases. However, the physiological role of SEC during development and homeostasis remains largely unexplored. Here we studied the function of SEC in adipogenesis by manipulating an essential scaffold protein AF4/FMR2 family member 4 (AFF4), which assembles and stabilizes SEC. Knockdown of AFF4 in human mesenchymal stem cells (hMSCs) and mouse 3T3-L1 preadipocytes inhibits cellular adipogenic differentiation. Overexpression of AFF4 enhances adipogenesis and ectopic adipose tissue formation. We further generate *Fabp4-cre* driven adipose-specific *Aff4* knockout mice and find that AFF4 deficiency impedes adipocyte development and white fat depot formation. Mechanistically, we discover AFF4 regulates autophagy during adipogenesis. AFF4 directly binds to autophagy-related protein ATG5 and ATG16L1, and promotes their transcription. Depleting ATG5 or ATG16L1 abrogates adipogenesis in AFF4-overepressing cells, while overexpression of ATG5 and ATG16L1 rescues the impaired adipogenesis in *Aff4*-knockout cells. Collectively, our results unveil the functional importance of AFF4 in regulating autophagy and adipogenic differentiation, which broaden our understanding of the transcriptional regulation of adipogenesis.

## Author summary

Obesity is a major health problem jeopardizing millions of individuals worldwide. From a pathological perspective, obesity occurs in the process of white adipose tissue expanding its mass through the enlargement of adipocyte size or advanced differentiation of adipocyte precursors to mature adipocytes. Studies have documented the dysregulated adipocyte metabolism of adipose tissue and associated disorders. However, our understanding of adipocyte development in which mesenchymal stem cells (MSCs) commit their fate and preadipocytes undergo differentiation and maturation is scarce. Here, we identify the super elongation complex (SEC) scaffold protein AFF4 as an essential regulator of adipogenesis. We reveal that AFF4 promotes adipocyte formation by regulating the cellular autophagic process. AFF4 directly regulates the transcription of the autophagy-related protein ATG5 and ATG16L1, which are essential for autophagosome formation. This finding further elucidates the physiological role of SEC during tissue development, besides its recognized role in cancer occurrence.

## Introduction

The prevalence of obesity is a major challenge to chronic disease prevention and intervention. The World Health Organization stated that there are more than one billion overweight adults around the world. As one of the major health problems, obesity has been identified as a key contributor to heart disease, type 2 diabetes, strokes, liver steatosis and other chronic diseases [1, 2]. Approximately 4 million deaths were caused by obesity annually and in 2015, high body mass index contributed to 4 million deaths globally, which represented 7.1% of the deaths from any cause [3, 4].

It is well recognized that obesity is closely related to genetic risk factors and unhealthy lifestyle such as calorie excess and lack of physical activity [5]. Pathogenesis of obesity is closely related with the process in which white adipose tissue (WAT) expands its mass through the enlargement of adipocyte size (hypertrophy) or advanced differentiation of adipose precursor cells (APCs) to mature adipocytes (hyperplasia) [6]. Studies have documented the dysregulated adipocyte metabolism, focusing on the pathologic hypertrophy of adipose tissue and associated disorders [6]. However, our understanding of early adipogenesis, namely the mesenchymal stem cells (MSCs) lineage commitment and APCs differentiation and maturation, is scarce [7].

The adipogenesis process is tightly controlled by systemic signals and transcriptional factors [7]. Systemic modulators are hormones and ligands such as insulin, glucocorticoid and bone morphogenetic proteins (BMPs). Important transcriptional regulators include the peroxisome proliferator-activated receptor gamma (PPARγ) and CCAAT/enhancer binding protein α (C/EBPα), which are necessary and sufficient to drive adipocyte differentiation [8]. Recently, accumulating transcription regulators have been reported to regulate adipogenesis. For instance, CBFA2T2 has been reported to facilitate H3K9me2 and H3K9me3 enrichment at CEBPA promoter, thus increasing cellular adipogenic differentiation [9]. SOX9 was found to maintain preadipocyte factor 1-expressing (Pref1^+^) adipocyte precursors and its inactivation is a prerequisite for adipogenic differentiation of Pref1^+^ cells into platelet-derived growth factor receptor α-expressing (PDGFRα^+^) cells [10]. Further illustration of the molecular mechanisms that govern MSCs and APCs differentiation into adipose depot may help developing therapeutic strategy for obesity.

The super elongation complex (SEC) consists of eleven-nineteen Lys-rich leukemia (ELL) family proteins, positive transcription elongation factor b (P-TEFb) and the mixed lineage leukemia (MLL) translocation partners, which is required for robust and productive gene transcription through the release of paused Pol II [11]. AF4/FMR2 family member 4 (AFF4) is the scaffold protein of SEC, which stabilizes the whole complex and enhances gene transcription efficiency [12]. To date, AFF4 was discovered to be closely relevant to mixed lineage leukemia and CHOPS syndrome [11, 13]. Furthermore, AFF4 played a role in tumorigenesis, osteogenesis and odontogenesis [14–17]. Previously, our work demonstrated AFF4 enhanced the osteogenic differentiation of human mesenchymal stem cells (hMSCs) *in vitro* and increased ectopic bone formation *in vivo* [15]. Nevertheless, the role of AFF4 in cellular adipogenic differentiation remains unknown. In this study, we explore the regulation of AFF4 on cellular adipogenesis and reveal an AFF4-dependent adipogenesis through autophagy.

## Results

### Depletion of AFF4 inhibits adipogenic differentiation

**Fig 1:**
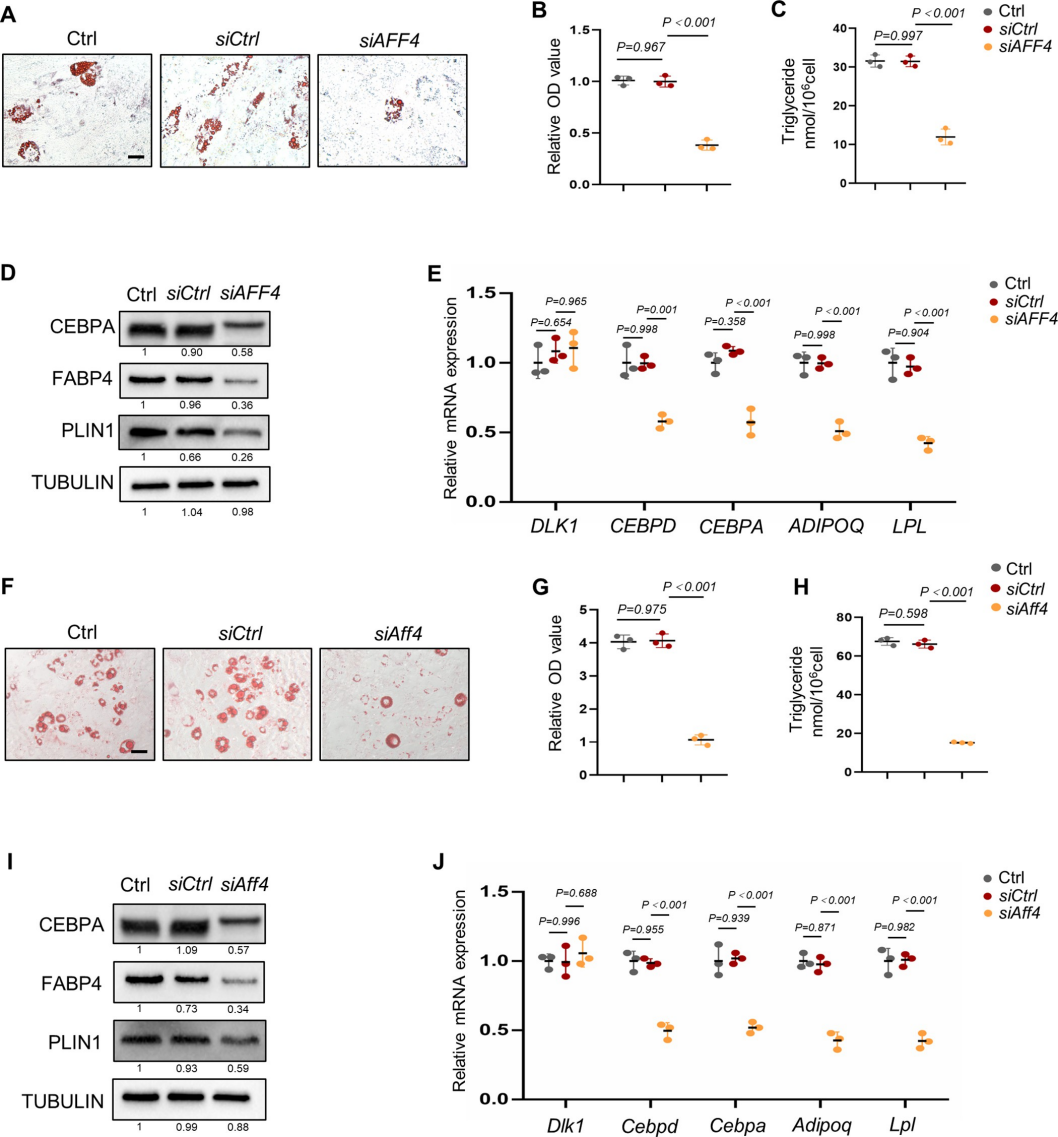
**Depletion of AFF4 inhibits adipogenic differentiation** (A-E) Adipogenic differentiation in AFF4-deficient hMSCs. (A) Representative images of oil red o staining. Scale bar, 25 μm. (B) Absorbance of lipids at 518 nm. n = 3. (C) Triglyceride quantification. n = 3. (D) Western blot analysis of the expression of adipogenic markers. (E) RT-qPCR of adipogenic markers. n = 3. (F-J) Adipogenic differentiation in AFF4-deficient 3T3-L1 cells. (F) Representative images of oil red o staining. Scale bar, 25 μm. (G) Absorbance of lipids at 518 nm. n = 3. (H) Triglyceride quantification. n = 3. (I) Western blot analysis of the expression of adipogenic markers. (J) RT-qPCR of adipogenic markers. n = 3. All quantified data are presented by as mean ± SD. The *P* values were calculated by one-way ANOVA with Tukey’s post hoc test.

To explore the potential function of AFF4 in cellular adipogenic differentiation, siRNA targeting AFF4 was used to knockdown AFF4 in hMSCs and 3T3-L1 preadipocytes. The knockdown efficiency was confirmed by quantitative real-time PCR (qPCR) and western blot (S1A, S1B, S1D and S1E Fig). Knockdown of AFF4 slightly inhibited cell proliferation (S1C and S1F Fig). Importantly, we found that depletion of AFF4 significantly impeded lipids and triglyceride accumulation in hMSCs (Fig 1A–1C). Depletion of AFF4 inhibited the expression of adipogenic markers, including CEBPA, fatty acid binding protein 4 (FABP4) and perilipin 1 (PLIN1) 3 days after adipogenic induction (Fig 1D). Additionally, qPCR analyses demonstrated that mRNA expression of adipogenic-related genes, *CCAAT/enhancer binding protein delta* (*CEBPD*), *CEBPA*, *Adiponectin* (*ADIPOQ*) and *lipoprotein lipase* (*LPL*) was significantly down-regulated upon AFF4 depletion in hMSCs (Fig 1E). We did not find a significant change of *delta-like 1* (*DLK1*) expression, an early negative adipogenic fate regulator, in AFF4-depleted cells during differentiation (Fig 1E). In agreement with the results of hMSCs, we observed adipogenic differentiation was impeded in *Aff4*-deficient 3T3-L1 cells (Fig 1F–1J).

### Overexpression of AFF4 increases adipogenesis

**Fig 2:**
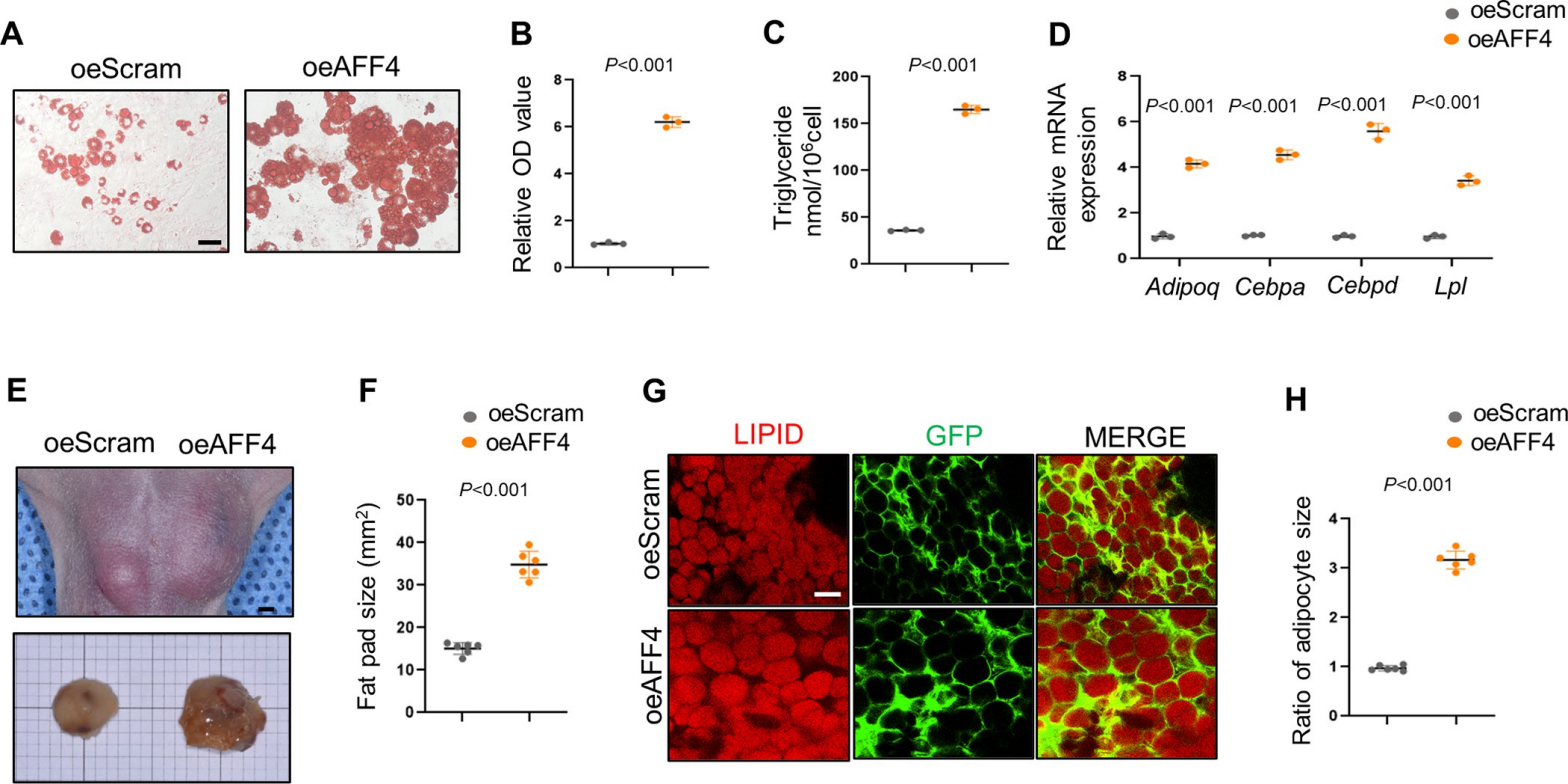
Overexpression of AFF4 enhances adipogenesis. (A-D) Adipogenesis in AFF4-overexpressing cells. (A) Representative images of oil red o staining of 3T3-L1 cells. (B) Quantification of oil red o staining, absorbance at 518 nm. n = 3. (C) Triglyceride quantification. n = 3. (D) RT-qPCR of adipogenic markers. n = 3. (E-H) Ectopic fat formation in nude mice. (E) Xenograft of oeScram and oeAFF4 3T3-L1 cells in nude mice and samples were collected after 6 weeks. Scale bar, 1mm. (F) Fat pad size quantification. n = 6. (G) Representative immunofluorescent images of lipids. Scale bar, 50 μm. (H) Adipocyte size quantification. n = 6. All quantified data are presented by as mean ± SD. The *P* values were calculated by two-tailed Student’s t-test.

Next, we generated a 3T3-L1 cell line stably expressing AFF4 using a lentivirus-based expression system. AFF4 expression efficiency was confirmed by western blot (S2A Fig) and qPCR (S2B Fig). As expected, overexpression of AFF4 significantly enhanced cell proliferation, cellular lipids and triglyceride accumulation (Figs S2C and 2A–2C). Consistently, the mRNA levels of adipocyte marker genes, including *Cebpd*, *Cebpa*, *Adipoq* and *Lpl*, were elevated in AFF4*-*overexpressing cells (Fig 2D). Ectopic transplantation of AFF4*-*overexpressing 3T3-L1 cells into immunodeficient nude mice formed larger size (mm^2^) of fat pads (Fig 2E and 2F) with an increased adipocyte size (Fig 2G and 2H).

### Conditional knockout of *Aff4* decreases fat mass in mice

**Fig 3:**
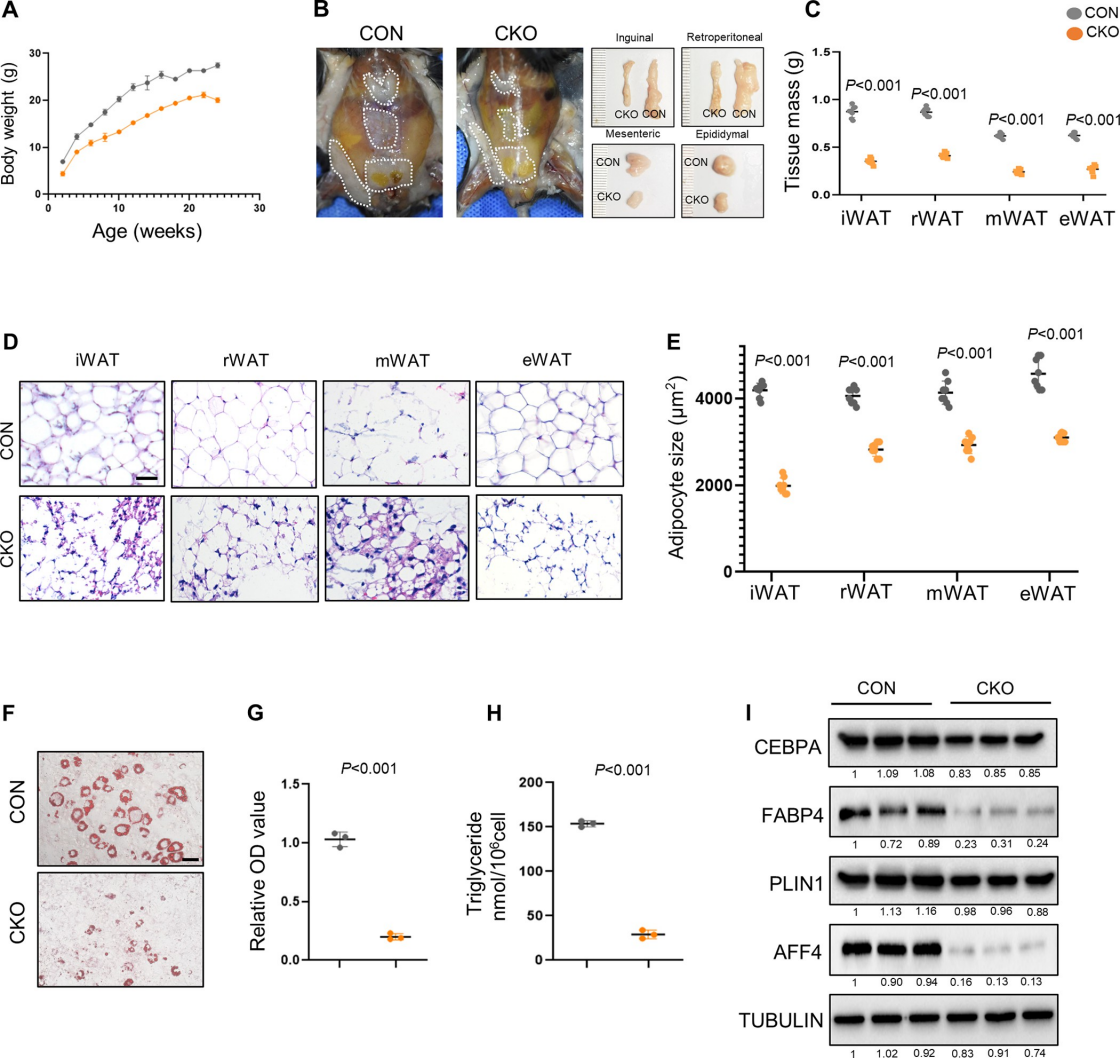
Conditional *Aff4* knockout decreases fat mass in mice. (A-E) WAT formation was impeded in *Aff4* knockout mice (A) Body weight of CON and CKO mice. n = 9. (B) Representative images of WAT from CON and CKO mice at 16 weeks of age. (C) Tissue weight of WAT is significantly lower in CKO mice. n = 8. (D) H&E staining of iWAT, rWAT, mWAT and eWAT in CON and CKO mice. Scale bar, 50 μm. (E) Adipocyte size quantification in WAT. n = 8. (F-I) Adipogenic differentiation of primary preadipocytes. (F) Representative images of oil red o staining. Scale bar, 25μm. (G) Quantitative analysis of oil red o staining, absorbance at 518 nm. n = 3. (H) Triglyceride quantification. n = 3. (I) Western blot of preadipocytes obtained from CON and CKO mice. Each lane corresponds to lysates from one mouse. All quantified data are presented by as mean ± SD. The *P* values were calculated by two-tailed Student’s t-test.

To further confirm the role of AFF4 in adipogenesis, we generated *Aff4* flox (*Aff4*^*fl/+*^) mice using CRISPR/Cas9 technique, and bred with *Fabp4-Cre* mice to conditionally delete *Aff4* (S3A and S3B Fig). *Fabp4-Cre; Aff4*^*fl/fl*^ (CKO) mice were viable and born at Mendelian’s ratio. CKO mice exhibited lower body weight at all examined time points from 2 weeks of age, compared with the control littermates (CON) (Fig 3A). The tissue weight of inguinal white adipose tissue (iWAT), retroperitoneal white adipose tissue (rWAT), mesenteric white adipose tissue (mWAT) and epididymal white adipose tissue (eWAT) of CKO mice was much lower than that of CON mice (Fig 3B and 3C). The WAT of CON mice exhibited a typical morphology that almost the entire cytosol was occupied by one large lipid droplet, while *Aff4* deficiency led to fibrosis and smaller adipocytes (Fig 3D and 3E).

Next, we cultured preadipocytes from WAT in CON and CKO mice. More lipids were detected in CON group after adipogenic differentiation (Fig 3F). Quantitative analysis of lipids and triglyceride further supported the incompetent adipogenesis of preadipocytes from CKO mice (Fig 3G and 3H). The decreased expression of CEBPA, FABP4 and PLIN1 revealed compromised adipogenic potential of preadipocytes from CKO mice (Fig 3I)

### AFF4 regulates adipogenic differentiation by targeting autophagy

**Fig 4:**
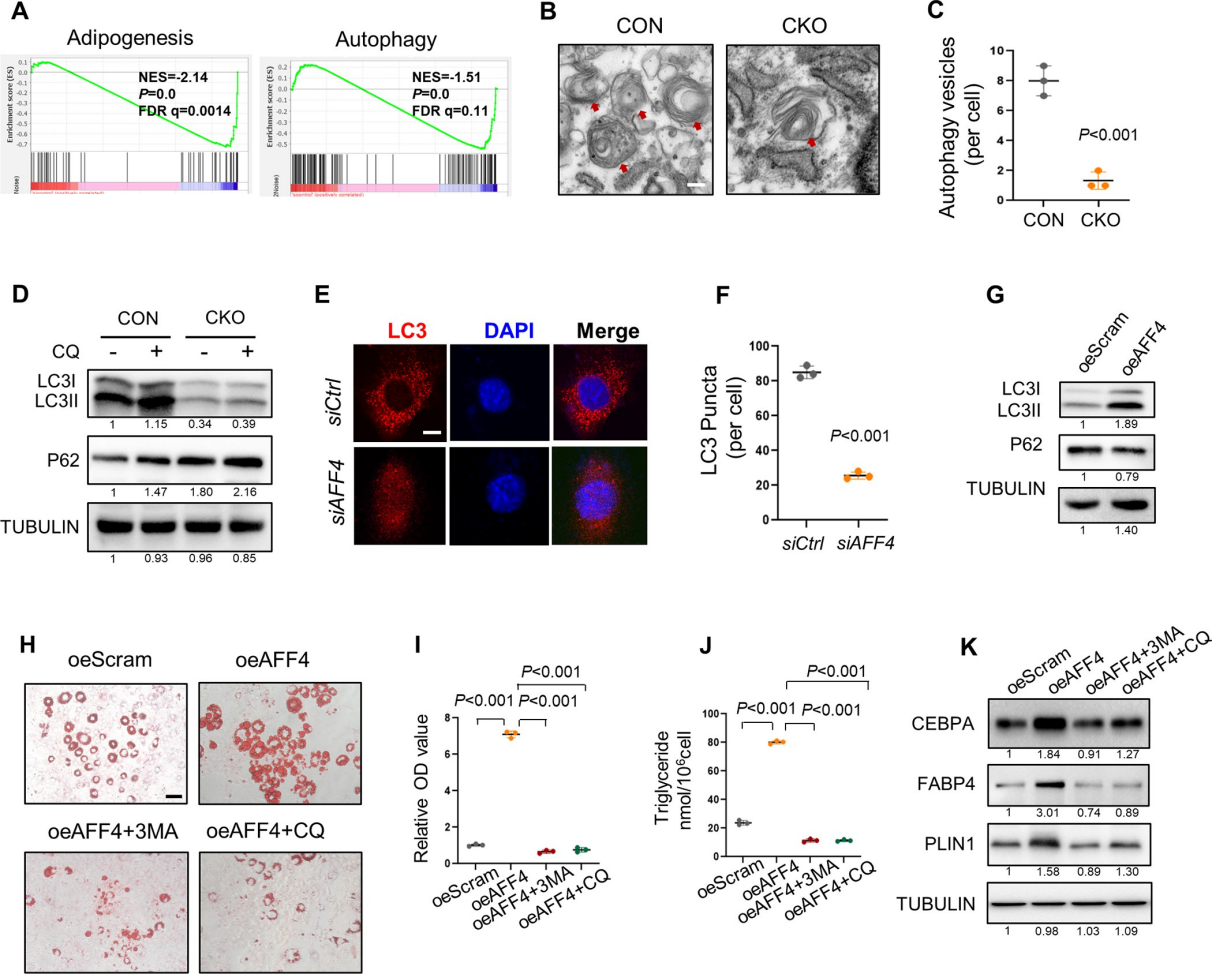
AFF4 controls adipogenesis via autophagy. (A) GSEA enrichment of adipogenesis and autophagy related pathway in AFF4-deficient hMSCs. (B, C) Representative TEM images and quantification of autophagy vesicles in preadipocytes from CON and CKO mice. Scale bar, 500 nm. n = 3. (D) Western blot analysis of autophagy-related proteins from primary preadipocytes of CON and CKO mice. CQ, chloroquine. (E, F) Representative immunofluorescent images and quantification of LC3 puncta in *siCtrl* and *siAFF4* hMSCs. Scale bar, 25 μm. n = 3. (G) Western blot analysis of autophagy related markers in AFF4 overexpressing and Ctrl 3T3-L1 cells. (H-K) Autophagy inhibitors corrected augmented adipogenic differentiation of AFF4-overexpressing cells. (H) Representative images of oil red o staining in the presence of 3-MA and CQ. Scale bar, 25μm. 3MA, 3-methyladenine. CQ, chloroquine. (I) Quantification of oil red o staining, absorbance at 518 nm. n = 3. (J) Quantification of triglyceride. n = 3. (K) Western blot of adipogenic markers in the presence of 3-MA and CQ. All quantified data are presented by as mean ± SD. The *P* values were calculated by two-tailed Student’s t-test (C, F) or one-way ANOVA followed by the Tukey’s post hoc test (I, J).

To investigate the mechanism, we performed RNA-seq in AFF4-deficient hMSCs. A total of 1316 genes were upregulated and 2272 genes were downregulated after AFF4 depletion. Gene set enrichment analysis confirmed the attenuated adipogenic differentiation after *AFF4* depletion and uncovered a remarkable down-regulation in autophagy related pathway (Fig 4A). To validate this observation, we analyzed autophagosome formation in preadipocytes obtained from CON and CKO mice by transmission electron microscopy (TEM). Deletion of *Aff4* reduced the number of normal autophagosomes and led to abnormally folded autophagosomes, indicating impaired activation of autophagy (Fig 4B and 4C). Consistently, western blot analysis showed a reduced LC3-II level in *Aff4* knockout cells (Fig 4D). To rule out that LC3-II reduction is an outcome of enhanced autophagic flux, we treated cells with a lysosome inhibitor chloroquine (CQ). The decreased expression of LC3-II in AFF4*-*deficient cells was not significantly changed after CQ treatment (Figs 4D and S4A). Immunofluorescence and western blot analyses further confirmed that the expression of LC3-II was correspondently altered when AFF4 was depleted or overexpressed (Figs 4E–4G and S4A, S5B). Mitochondria number was significantly increased in CKO preadipocytes (S4B–S4D Fig). On the other hand, we did not observe an ER retention in CKO preadipocytes (S4E–S4G Fig).

Next, we treated AFF4-overexpressing cells with autophagy inhibitor 3-methyladenine (3MA) or CQ and investigated the adipogenic capacity. Both 3MA and CQ reversed the augmented lipids formation and triglyceride accumulation in AFF4-overexpressing cells (Fig 4H–4J). The expression of CEBPA, FABP4 and PLIN1 in AFF4-overexpressing cells was corrected as well (Fig 4K).

### AFF4 targets ATG5 and ATG16L1

**Fig 5:**
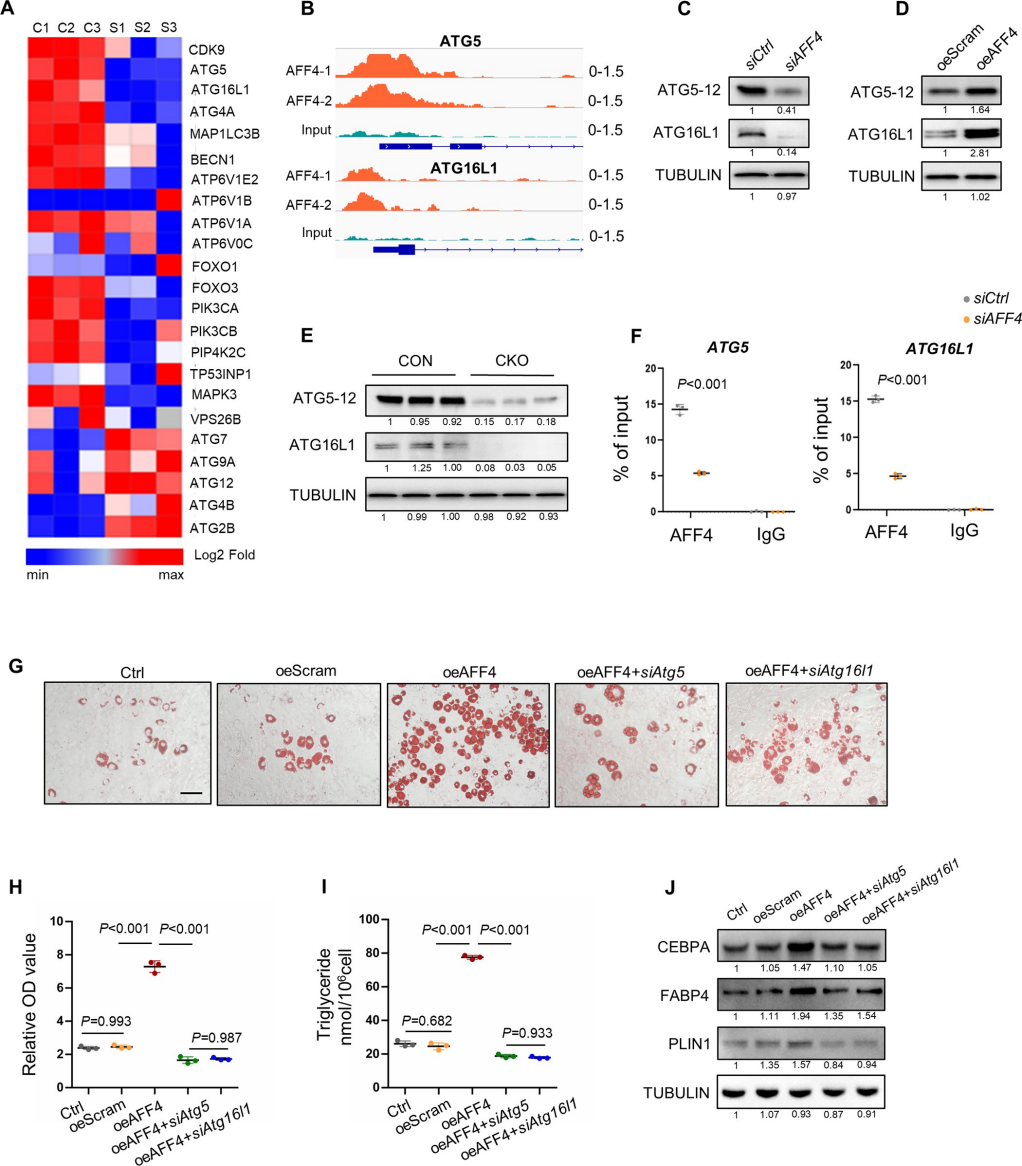
AFF4 regulates autophagy by targeting ATG5 and ATG16L1. (A) Heatmap of autophagy related genes in *siCtrl* and *siAFF4* hMSCs. (B) ChIP-Seq analysis of AFF4 association on the promotor of *ATG5* and *ATG16L1*. (C-E) Western blot analysis of ATG5-12 and ATG16L1 expression in AFF4 depleted hMSCs (C), AFF4 overexpressing 3T3-L1 cells (D) and preadipocytes from CON and CKO mice (E). (F) ChIP-qPCR showed AFF4 binds to the promoter region of *ATG5* and *ATG16L1*. n = 3. (G-J) Silencing of *Atg5* or *Atg16l1* corrected enhanced adipogenesis of AFF4-overexpressing 3T3-L1 cells. (G) Representative images of oil red o staining. Scale bar, 25 μm. (H) Quantitative analysis of oil red o staining. n = 3. (I) Triglyceride quantification. n = 3. (J) Western blot analysis of adipogenesis-related proteins. All quantified data are presented by as mean ± SD. The *P* values were calculated by two-tailed Student’s t-test (F) or one-way ANOVA followed by the Tukey’s post hoc test (H, I).

The process of autophagy involves sequential steps to form phagophore, autophagosome and autolysosome. Autophagy related (ATG) protein family is one of the most important proteins to assist autophagosome formation [18]. Therefore, we sought to explore whether AFF4 could directly regulate the expression of the ATG family members. We initially analyzed the gene expression profile in our RNA-seq data and found that expression of *ATG5*, *ATG16L1 and ATG4A* was significantly reduced after *AFF4* depletion, while that of *ATG7*, *ATG9A*, *ATG12*, *ATG4B and ATG2B* was upregulated (Fig 5A). We then explored into published AFF4 ChIP-seq data and unveiled that AFF4 was highly enriched on the promotor of *ATG5* and *ATG16L1* (Fig 5B).

The levels of AFF4, ATG5 and ATG16L1 were all upregulated during adipogenic differentiation (S5A Fig). More importantly, western blot analysis confirmed that ATG5-12 and ATG16L1 expression was down-regulated when AFF4 was depleted (Figs 5C and S5B). In AFF4-overexpressing cells, we obtained the opposite results (Fig 5D). The reduction of ATG5 and ATG16L1 expression was further verified in preadipocytes isolated from CON and CKO mice (Fig 5E). Next, we performed ChIP-qPCR assay to test whether AFF4 directly regulates the transcription of *ATG5* and *ATG16L1*. Notably, the enrichment of AFF4 at the promoter of *ATG5* and *ATG16L1* was significantly decreased in response to the depletion of AFF4 (Fig 5F). We further explored whether silencing ATG5 or ATG16L1 reversed the enhanced adipogenesis in AFF4-overexpressing 3T3-L1 preadipocytes. Knockdown of *Atg5* or *Atg16l1* was able to abolish the adipogenic differentiation in AFF4-overexpressing cells (Fig 5G–5J).

### Overexpression of ATG5 and ATG16L1 rescues adipogenic potential of *Aff4*-deficient cells

**Fig 6:**
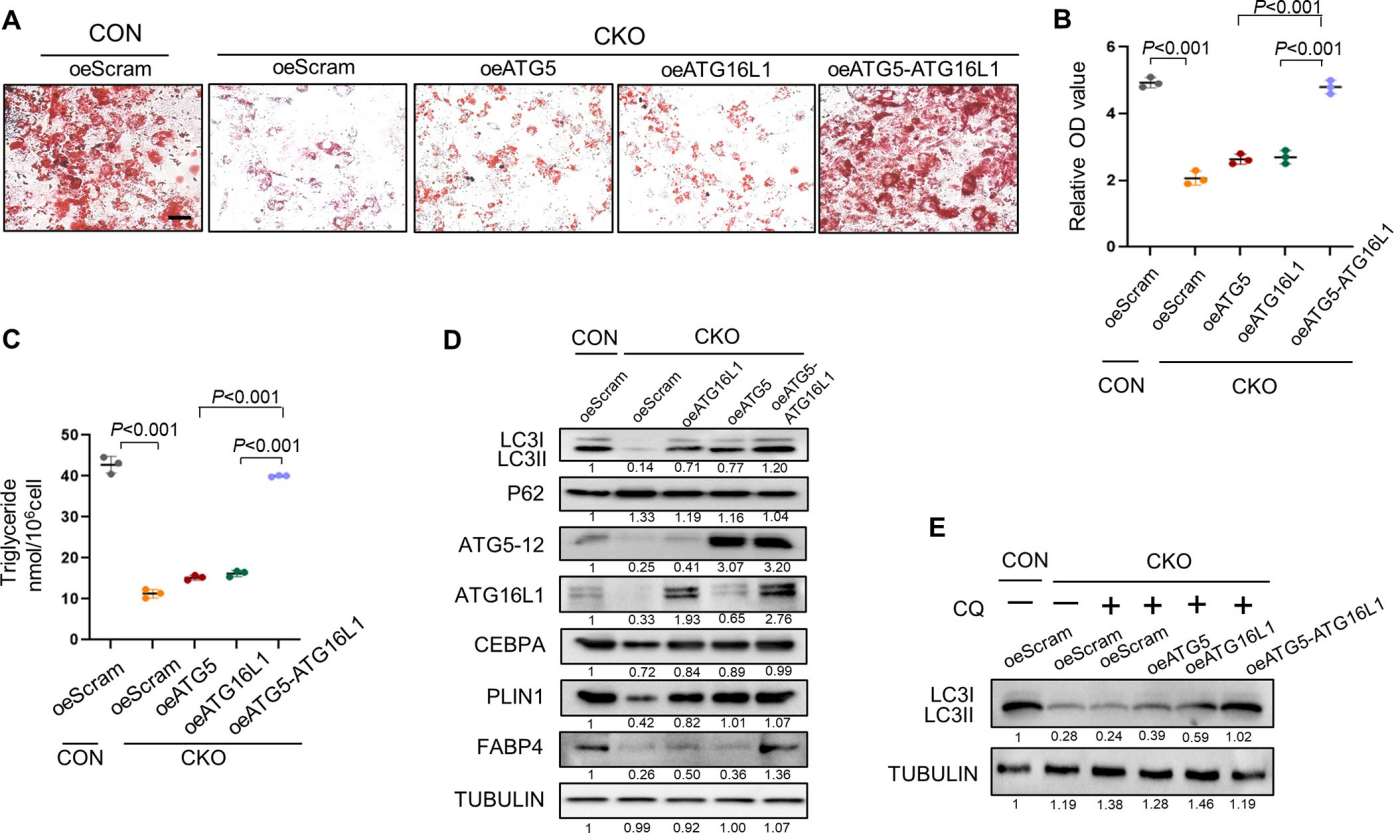
Overexpression of ATG5 and ATG16L1 rescues adipogenic potential of *Aff4*-deficient cells. (A, B) Representative images and quantification of oil red O staining of CON and CKO preadipocytes overexpressing ATG5 or ATG16L1. Scale bar, 25μm. n = 3. (C) Triglyceride quantification. n = 3. (D) Western blot analysis of autophagy related markers and adipogenic markers. (E) Western blot analysis of LC3. CQ, chloroquine.

We thus isolated preadipocytes from CON and CKO mice and modulated ATG5 or ATG16L1 expression by lentivirus transfection. Overexpression of ATG5 or ATG16L1 alone hardly rescued the adipogenic differentiation, lipids formation or triglyceride accumulation, while simultaneous overexpression of ATG5 and ATG16L1 greatly rescued adipogenesis (Fig 6A–6D).

We then evaluated the effect of ATG5 and ATG16L1 overexpression on autophagy. Simultaneous overexpression of ATG5 and ATG16L1 rescued the diminished autophagy activity in preadipocytes from CKO mice (Fig 6D). CQ treatment led to accumulation of LC3-II upon ATG5 and ATG16L1 overexpression, further testifying the rescue of autophagy (Fig 6E).

## Discussion

The cellular component of a mature WAT cell is a large lipid droplet with the nucleus squeezed aside, surrounded by a thin layer of cytoplasm [6]. The lipid droplets can extend to more than 100 μm under advanced adipocyte hypertrophy [19]. To differentiate into mature adipocytes, fibroblast-like progenitors not only undergo dramatic morphological changes, but also different stages of fate commitment and differentiation [7]. In the present study, we selected multipotent hMSCs and lineage committed 3T3-L1 preadipocytes, to comprehensively explore the regulation of AFF4 in adipogenesis.

Adipogenic differentiation can be roughly divided into early and late stages. At the early stage, activation of PPARγ and CEBPs family proteins initiates the cascade of adipogenic differentiation [20]. The expression of FABP4, ADIPOQ, LPL and PLIN1 can reflect the formation and maturation of lipid droplets [7, 21]. We observed that knock-down of AFF4 attenuated adipogenic differentiation as revealed by oil red o staining. We showed by qPCR and western blot assay that the expression of early adipogenic markers CEBPA and CEBPD, and late markers FABP4, ADIPOQ, LPL and PLIN1 is significantly downregulated. At the same time, ectopic implantation of AFF4*-*overexpressing preadipocytes to immunodeficient nude mice resulted in more and larger adipose tissue formation.

Based on these data, we further generated *Fabp4-Cre* mediated *Aff4* conditional knockout mice. *Aff4* CKO mice showed an obvious lower body weight and formed less WAT. The adipose tissue of *Aff4* CKO mice was less differentiated, and the adipogenic markers expressed by primary adipocytes were significantly reduced. Together, these data illustrated AFF4 regulated white adipose tissue formation by controlling cellular adipogenic differentiation.

FABP4, also known as Adipocyte Protein 2 (AP2), is a member of the FABPs family and plays an important role in adipose tissue differentiation and lipid metabolism [22–24]. Using lineage tracing, researchers demonstrated that FABP4 was expressed in adipose precursor cells in stromal vascular fraction (SVF) of WAT and brown adipose tissue [25]. Therefore, *Fabp4-Cre* mice have been widely used for study of white fat tissue development and maturation [22, 26, 27]. Another genetic mouse model widely used in adipocyte research is *Adipoq-Cre* [28]. Considering ADIPOQ is exclusively expressed in mature adipocytes, we chose *Fabp4-Cre* to study AFF4 on adipose progenitor differentiation.

Adipogenesis is regulated by a variety of signal pathways such as the insulin signaling, glucocorticoid signaling and BMP signaling [7, 23]. Autophagy is also tightly associated with cellular adipogenesis, as organelles of MSCs and preadipocytes must undergo gradual degradation by autophagy to form adipocytes. Autophagy begins from the formation of the membrane-like structures in the cytoplasm and these membrane-like structures continue to form phagophore and then autophagosome, which fuses into lysosome for degradation [18]. ATG family, especially ATG5 and ATG7, plays pivotal roles in autophagy vesicle membrane elongation [18]. ATG5 forms a conjugated complex with ATG12 and is involved in the autophagy vesicle membrane formation and extension [18]. ATG7 acts like as a ubiquitin E1-like activating enzyme and is required for ATG5 and ATG12 conjugation [29]. ATG16 has two structural subtypes, ATG16L1 and ATG16L2, and ATG16L1 was closely related to the formation of autophagy vesicles [30]. ATG5-12 complex can form hexamer complex with ATG16L1 in cytoplasm, which continuously recruits LC3 to the membrane surface to form complete autophagy vesicle structure [29].

Several studies reported the regulation of autophagy in adipogenesis and proposed that autophagy aided in adipogenesis by degrading cellular organelles [31–33]. During adipogenic differentiation of mouse embryonic fibroblasts (MEFs), LC3-II was up-regulated along with the time of adipogenic induction [31]. *Atg5*^-/-^ MEFs failed to form lipid droplets and gradually turned apoptotic after 3 days of adipogenic induction, suggesting that *Atg5* was involved in the early stages of adipogenic differentiation. Indeed, treatment of CQ to MEFs alone inhibited adipogeneis [31]. Another study reported that the *Fabp4-Cre;Atg7*^*fl*^*/*^*fl*^ mice were smaller and thinner than the control mice but with similar food intake. The total fat was decreased by about 30% compared with control mice [32]. Collectively, these data suggests when autophagy is impeded, adipogenesis could be attenuated from early stage. In the present study, combining the ChIP-seq and RNA-seq data and together with gain-of-function/loss-of-function analysis, we verified that expression of AFF4 was correlated with the transcription of ATG5 and ATG16L1. Incompetent transcription of ATG5 and ATG16L1 after AFF4 depletion impeded the autophagosome formation, which led to inefficient degradation of cellular components, and in this way the adipogenesis was impaired. We did not detect a direct regulation of AFF4 on other ATG genes, and the upregulation of certain ATG genes in AFF4-depleted cells, such as ATG7, ATG9A and ATG12, is possibly secondary and compensatory to the changes of autophagy.

On account of the close relationship of adipogenesis and mitochondria, we further examined the mitochondria after AFF4 knockout. The number of mitochondria was increased in preadipocytes of CKO mice (S4B–S4D Fig). A similar increase of mitochondria number was observed in *Fabp4-Cre;Atg7*^*fl/fl*^ mice as well [32]. We thus suppose mitochondria upregulation is a result of impeded autophagy in AFF4-deficient cells. However, thorough metabolic profile of CKO mice remained to be further clarified.

As the scaffold protein of SEC, AFF4 contributes to the stability of SEC. Lin et al. found AFF4 was required for the association of CDK9 and cyclin T1 with SEC [12]. Another study also suggests AFF4 mediates the interaction between ELL2 and P-TEFb. The SEC component ELL2 was found short-lived and required AFF4 to maintain its stability [34]. In this study, we evaluated the bulk protein level of the SEC components by western blot. In accordance with previous reports, we detected lower levels of ELL1 and ELL2, but not EAF1 and cyclin T1 (CCNT1) in CKO preadipocytes (S6 Fig). Surprisingly, we found a reduced protein level of CDK9, which was reported unchanged by Lin et al [12]. As the mRNA level of CDK9 was also reduced in our RNA-seq analysis, we presume the change of CDK9 expression is regulated at the transcription level when AFF4 was depleted. Therefore, further study regarding the relation between AFF4 and CDK9 is needed.

AFF4 functions in development besides its important role in cancer and virus infection [11, 35]. Missense mutation in human AFF4 gene leads to CHOPS syndrome that phenotypically overlaps Cornelia de Lange syndrome (CdLS) [13]. Urano et al. found that most whole body *Aff4-*knockout mice died in utero, with alveoli development severely impaired. The 13% mice survived exhibited an infertility with defective spermiogenesis [36]. To our best knowledge, the present study is the first work reporting conditional knockout of *Aff4* in specific tissue.

One limitation of this study is the lack of clarification of AFF4 function in SEC and the promoter-proximal paused Pol II release. Though we verified a direct regulation of AFF4 on ATG5 and ATG16L1, whether and how AFF4 regulates the recruitment of P-TEFb to their promoters need to be further explored. Besides, the metabolic prolife of *Aff4*-CKO mice is largely unexplored. Further study illustrating the regulation of AFF4 on adipose lipid and energy metabolism, and related diseases like diabetes, is warranted.

In summary, our present study unveils the unknown function of AFF4 in adipogenesis and its intriguing mechanism by targeting autophagy through ATG5 and ATG16L1. This study broadens the knowledge of transcriptional regulation of adipogenesis and might provide insights into diseases such as obesity and lipodystrophy.

## Methods

### Ethics statement

All animal experiments were approved by and performed under the guidance of the Subcommittee on Research and Animal Care (SRAC) of Sichuan University.

Primers for qPCR and genotyping were listed in S1 Table. Details of reagents and resources were listed in S2 Table.

### Animals

Nude mice (6 weeks of age) were purchased from the Chengdu Dossy Experimental Animals CO. LTD. *Aff4*^*fl/+*^ mice were generated by Biocytogen company (Beijing, China). *Fabp4-*Cre transgenic mice were purchased from Jackson Laboratory (strain number 018965). Mice were maintained at 23 ± 2°C with a humidity of 35 ± 5% under a 12 h light and 12 h dark cycle, with ad libitum access to water and food. Body weight was measured every week after 2 weeks of age.

### Cells

The hMSCs were obtained from Cyagen (Guangzhou, China) and 3T3-L1 were obtained from ATCC (Virginia, United States) and tested negative for mycoplasma contamination before use. hMSCs were cultured in Minimum Essential Medium α and 3T3-L1 cells were in high-glucose Dulbecco’s Modified Eagle Medium containing 10% fetal bovine serum and 1% penicillin-streptomycin. Adipogenic differentiation medium contains 0.5 μM IBMX,1 μM dexamethasone and 10 μg/mL insulin. For hMSCs and 3T3-L1 cells, adipogenic differentiation lasted for 14 days to form mature adipocytes [37]. All medium was changed every 2 days thereafter. All cells were maintained at 37°C in a humidified 5% CO_2_ incubator.

### Isolation of mice preadipocytes

White adipose tissue was harvested from CON and CKO mice (8 weeks of age) and then treated with type I collagenase for 30 min at 37°C [38]. The digested tissue was then filtered through a 100 μm filter mesh and centrifuged at 1500 rpm for 5 min. Cells were then resuspended and cultured in DMEM containing 20% FBS.

### siRNA knockdown and lentivirus-mediated overexpression

Small interfering RNAs (siRNAs) targeting AFF4 were purchased from Santa Cruz (human *siAFF4*, sc-91842; mouse *siAff4*, sc-140897). Each siRNA consists of mixes of three to five target-specific 19- to 25- nucleotide siRNAs. siRNAs targeting ATG5 and ATG16L1 were purchased from Sangon (*siAtg5*, sense: *CCUUGGAACAUCACAGUACAUTT*, antisense: *AUGUACUGUGAUGUUCCAAGGTT*; *siAtg16l1*, sense: *CCUUCUGGAUUCUAUCACUAATT*, antisense: *UUAGUGAUAGAAUCCAGAAGGTT*). For siRNA mediated knockdown, cells were transfected using Lipofectamine RNAimax as previously reported [39]. Briefly, for one well of 6-well plate, 1 μg siRNA duplex was diluted into 100 μL Opti-MEM I (solution A) and 5 μL RNAimax was diluted into 100 μL Opti-MEM I (solution B). Then, solution A was mixed to solution B and incubated for 15 min at RT. The mixture was then added to 800 μL growth medium without antibiotics. The medium was incubated overnight and changed to fresh growth medium with antibiotics the next day. The knockdown efficiency was examined by western blot and RT-qPCR after 48 hours of transfection.

For overexpression, the lentivirus vectors were obtained from GeneChem (oeAFF4, oeATG5, oeATG16L1). Cells transfected with an empty GV358 vectors (Ubi-MCS-3FLAG-SV40-EGFP-IRES-puromycin) were used as control [37]. Cells were cultured in 6-well to 30% confluence and transfected with lentivirus at an MOI = 20. The lentivirus along with 1× Hitrans G Polybrene were added into 1mL growth medium for 12 hours. Cells were then treated with puromycin for 7–10 days to obtain stable cell clones. The efficiency of transfection was confirmed by western blot and RT-qPCR.

### Oil Red O staining

Cells were washed and fixed with 4% paraformaldehyde for 20 min. After washed with PBS, the cells were treated with 60% isopropanol and stained with Oil Red O for 20 min, then rinsed three times with distilled water and observed under a microscope [40]. For quantification, Oil Red O-stained lipids were eluted in 100% isopropanol, and the optical density (OD) was measured at 518 nm.

### Measurement of triglyceride levels

For triglyceride measurement, differentiated cells were washed twice with PBS and lysed by lysis buffer. The triglyceride levels of the cells were measured using the triglyceride assay kit according to the manufacturers’ protocol.

### Western blot assay

Cells and tissues were lysed on ice using protein extraction kit with protease and phosphatase inhibitor cocktail (Sabbiotech, PE001) for 20 min. The protein concentration was determined using the BCA protein assay kit (Beyotime, P0012). Proteins were separated by SDS-PAGE and then transferred to polyvinylidene difluoride membrane (Millipore, ISEQ00010) membranes. The membranes were blocked with 5% non-fat milk at room temperature for 1 h and then incubated with corresponding primary antibodies overnight at 4°C. Next day, the membranes were incubated with goat anti-rabbit HRP-conjugated secondary antibodies at a dilution of 1:5,000 at room temperature for 1 h.

### Quantitative reverse transcription PCR (RT-qPCR) assay

Total RNA was extracted using TRIzol reagent and reverse transcribed to cDNA using PrimeScript RT reagent Kit with gDNA Eraser. qPCR analysis was performed using the iTaq Universal SYBR Green PCR Supermix. The data was analyzed following the 2^-ΔΔCt^ method and calculated using GAPDH as the normalization control (S1 Table).

### Cell viability assay

Cell viability was assessed using the Cell Counting Kit-8 (CCK8).10 μL CCK8 was added to each well of 96-well plate and incubated at 37°C for 4 h. The absorbance value of each well was detected by a microplate reader at 450 nm.

### Ectopic fat formation

Approximately 2 ×10^5^ cells overexpressing AFF4 or empty vector GFP were mixed with 50% matrigel suspensions. The mixture was injected subcutaneously flanking the sternum of the nude mice. Mice were then subject to high-fat diet. Six weeks after transplantation, the transplants were collected and fixed with 4% paraformaldehyde, and then dehydrated and embedded in optical cutting temperature (OCT) compound for tissue section.

### Nile red staining

Frozen tissue was sectioned into slices with thickness of 20 μm (CM3050S; Leica) before staining. The slices were permeabilized with Triton X-100 for 10 min and stained with Nile Red solution (1:2000) for 15 minutes and then DAPI for 5 min at 37°C. Images were captured by confocal microscope (Olympus, FV3000).

### Hematoxylin and eosin (H&E) staining

iWAT, rWAT, mWAT and eWAT obtained from CON and CKO mice were fixed in 4% paraformaldehyde for 24 h at 4°C. Then the WAT was dehydrated and embedded in paraffin. Tissue was sectioned into slices with thickness of 5 μm. The slices were stained and observed under light microscope.

### Immunofluorescence analysis

Cells were washed with PBS and fixed with 4% paraformaldehyde for 20 min at room temperature, and then permeabilized with Triton X-100 for 10 min. After blocking with 5% BSA for 1h, cells were incubated with primary antibodies at 4°C overnight. The next day, cells were treated with the goat anti-rabbit Alexa Fluor 550 secondary antibodies for 1 h. Nuclei were stained with DAPI for 5 min at room temperature. Images were taken by a confocal laser microscope (Olympus, FV3000).RNA-Seq

RNA-Seq libraries were prepared using the Illumina TrueSeq mRNA sample preparation kit according to the manufacturer’s instruction and single-end sequenced on an Illumina HiSeq 3000 machine. We used FastQC (v0.11.5) to control the quality of RNA-Seq and aligned the reads to hg19 genome using HISAT2 (v.0.0.5). Gene expression level was considered significantly changed if log_2_FC (Fold change) > 1 or < -1, with adjusted *P* value < 0.05 [41]. Gene Set Enrichment Analysis (GSEA) for the related pathway was performed using GSEA software. Heat-map in this study was drawn using heatmap package in R 3.6.1.

### Transmission electron microscopy (TEM)

Cells were washed with PBS and fixed in 2.5% glutaraldehyde for 4h, and then fixed with 1% osmium tetroxide (OsO_4_) for 2 h. The samples were dehydrated with increasing concentrations of ethanol (30%, 50%, 70%, 80%, 90%, 95% and 100%) and transferred to acetone for 20 min. After placing in 1:1 mixture of absolute acetone and the final Spurr resin (SPI-CHEM) mixture for 1h at room temperature, the samples were transferred to 1:3 mixture of absolute acetone and the final resin mixture for 3 h and to final Spurr resin mixture overnight. The ultra-structures of cells were observed under a transmission electron microscope.

### Chromatin immunoprecipitation assay

Chromatin immunoprecipitation (ChIP) assays were performed utilizing EZ-Zyme Chromatin Prep Kit and EZ-Magna ChIP HiSens Chromatin Immunoprecipitation Kit as previously described [42]. The antibodies used for ChIP assay were anti-AFF4 (#A302-538A, Bethyl) and IgG (#CS200581, Millipore). qPCR was performed to quantify the precipitated DNA samples. Data was shown as the expression percentage of input DNA.

### Statistical analysis

All values were expressed as mean ± SD. For normality test, Shapiro-Wilk test was applied using SPSS software. Differences were evaluated by two-tailed Student’s t-test for comparison between two groups or by one-way analysis of variance (ANOVA) followed by the Tukey’s post hoc test for multiple comparisons. A *P* value of less than 0.05 was considered statistically significant.

## Supporting information

S1 Fig Depletion of AFF4 in hMSCs and 3T3-L1 cells. (A, B) Western blot and RT-qPCR analysis of AFF4 expression 48 h after *AFF4* knockdown in hMSCs. (C) CCK8 assay of hMSCs proliferation. n = 6. (D, E) Western blot and RT-qPCR analysis of AFF4 expression 48 h after *Aff4* knockdown in 3T3-L1 cells. (F) CCK8 assay of 3T3-L1 cells proliferation. n = 6. All quantified data are presented by as mean ± SD. The *P* values were calculated by one-way ANOVA followed by the Tukey’s post hoc test.
(TIF)

S2 Fig Overexpression of AFF4 in 3T3-L1 cells. (A, B) Western blot and RT-qPCR analysis of AFF4 expression in Ctrl, oeScram and oeAFF4 in 3T3-L1 cells. (C) CCK8 assay of 3T3-L1 cells proliferation. n = 6. All quantified data are presented by as mean ± SD. The *P* values were calculated by one-way ANOVA followed by the Tukey’s post hoc test.
(TIF)

S3 Fig Construction of *Aff4* conditional knockout mice. (A) Schematic representation of *Aff4* conditional knockout strategy. The exon 2 and exon 3 are deleted after Cre-mediated recombination. (B) Representative images of mice genotyping.
(TIF)

S4 Fig The detection of autophagy, mitochondria and endoplasmic reticulum. (A) Western blot analysis of autophagy related markers in *siCtrl* and *siAFF4* hMSCs. CQ, chloroquine. (B, C) Representative images and quantification of mitochondria in preadipocytes. n = 6. (D) TEM of preadipocytes from CON and CKO mice, yellow arrow indicating mitochondria. Scale bar, 5 μm. (E, F) Representative images and quantification of endoplasmic reticulum. n = 6. (G) TEM of preadipocytes from CON and CKO mice, yellow arrow indicating endoplasmic reticulum. Scale bar, 5 μm. All quantified data are presented by as mean ± SD. The *P* values were calculated by two-tailed Student’s t-test.
(TIF)

S5 Fig AFF4 targets ATG5 and ATG16L1. (A) Western blot analysis of AFF4, ATG5 and ATG16L1 expression during adipogenic differentiation of 3T3-L1 cells for 0, 1, 3, 5 and 7 days. (B) Western blot analysis of autophagy related markers in *siCtrl* and *siAff4* 3T3-L1 cells after adipogenic differentiation for 3, 5 and 7 days.
(TIF)

S6 Fig Western blot of SEC components derived from preadipocytes bulk protein lysates. (TIF)

S1 Table Primers for RT-qPCR and genotyping. (DOCX)

S2 Table Reagents and resource. (DOCX)
