# Supplementary figures and images for: AFF4 regulates cellular adipogenic differentiation via targeting autophagy
Source: PLoS Genet. 2022 Sep 23;18(9):e1010425. doi: 10.1371/journal.pgen.1010425 (PMC9534390; doi:10.1371/journal.pgen.1010425)

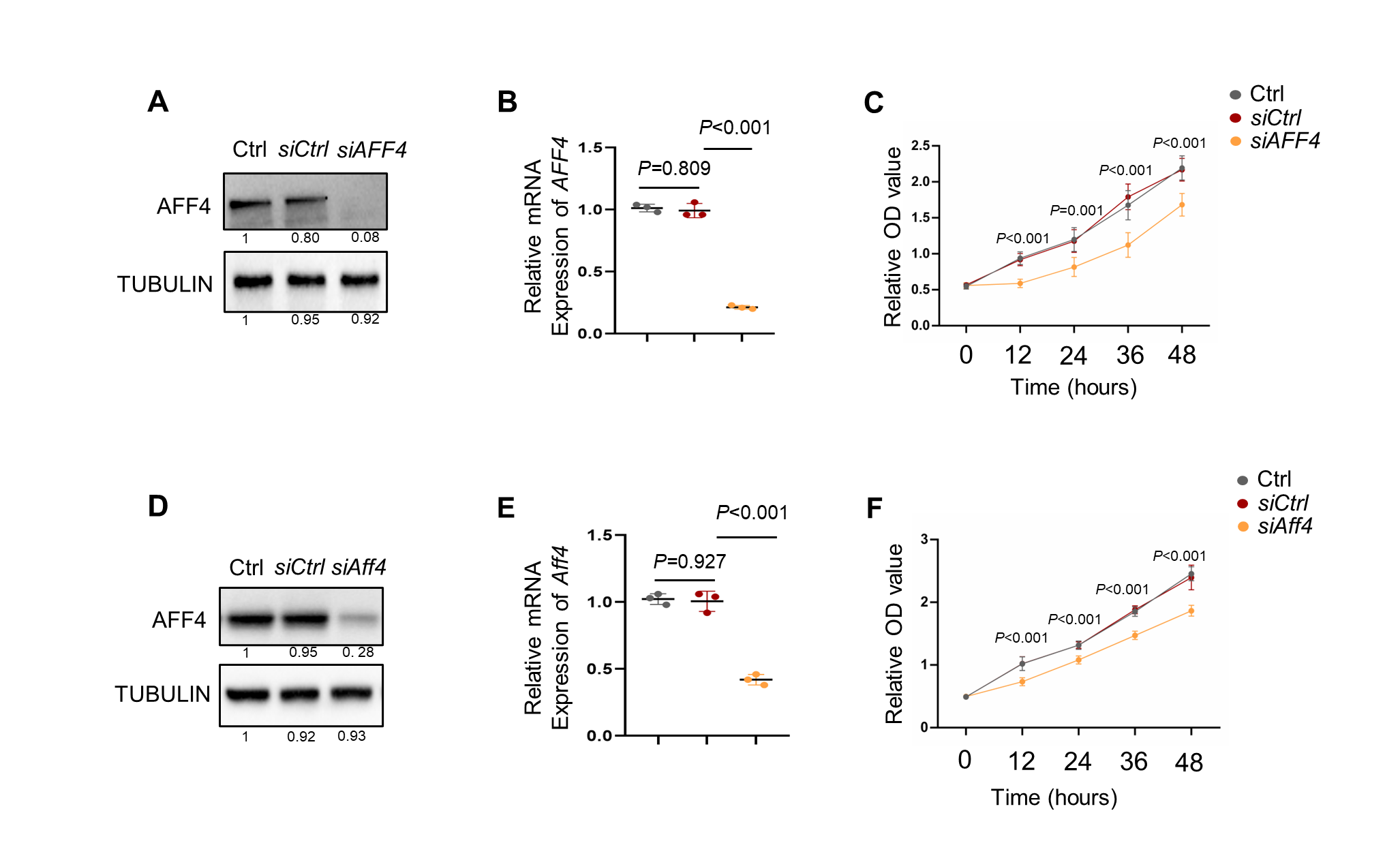

Supplement: S1 Fig — (A, B) Western blot and RT-qPCR analysis of AFF4 expression 48 h after AFF4 knockdown in hMSCs. (C) CCK8 assay of hMSCs proliferation. n = 6. (D, E) Western blot and RT-qPCR analysis of AFF4 expression 48 h after Aff4 knockdown in 3T3-L1 cells. (F) CCK8 assay of 3T3-L1 cells proliferation. n = 6. All quantified data are presented by as mean ± SD. The P values were calculated by one-way ANOVA followed by the Tukey’s post hoc test. (TIF) [file pgen.1010425.s001.TIF]

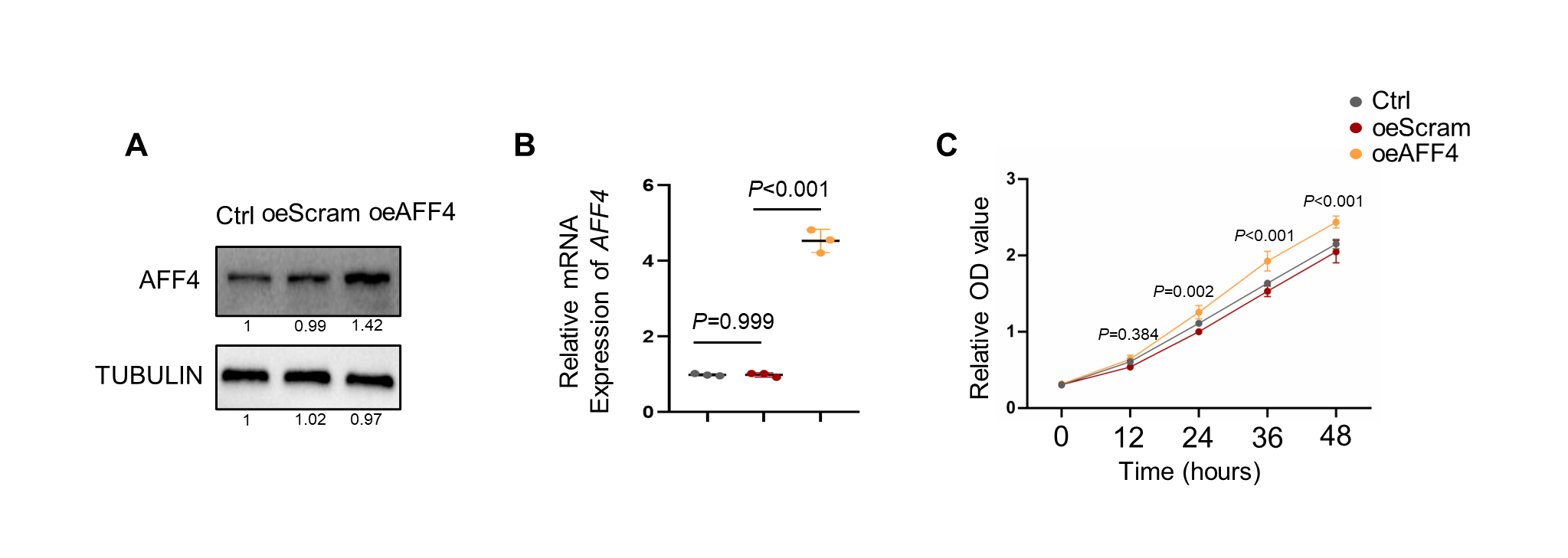

Supplement: S2 Fig — (A, B) Western blot and RT-qPCR analysis of AFF4 expression in Ctrl, oeScram and oeAFF4 in 3T3-L1 cells. (C) CCK8 assay of 3T3-L1 cells proliferation. n = 6. All quantified data are presented by as mean ± SD. The P values were calculated by one-way ANOVA followed by the Tukey’s post hoc test. (TIF) [file pgen.1010425.s002.TIF]

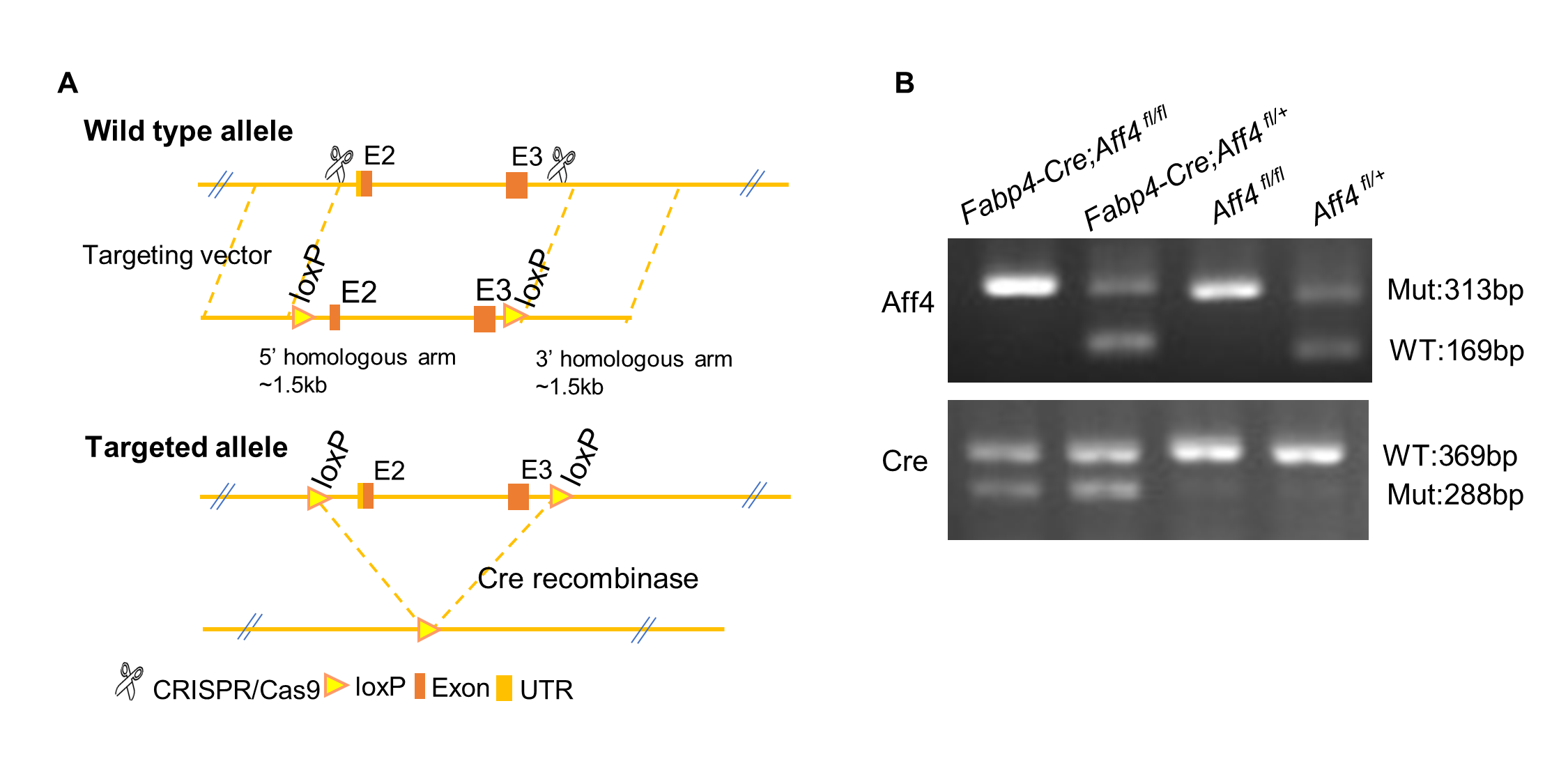

Supplement: S3 Fig — (A) Schematic representation of Aff4 conditional knockout strategy. The exon 2 and exon 3 are deleted after Cre-mediated recombination. (B) Representative images of mice genotyping. (TIF) [file pgen.1010425.s003.TIF]

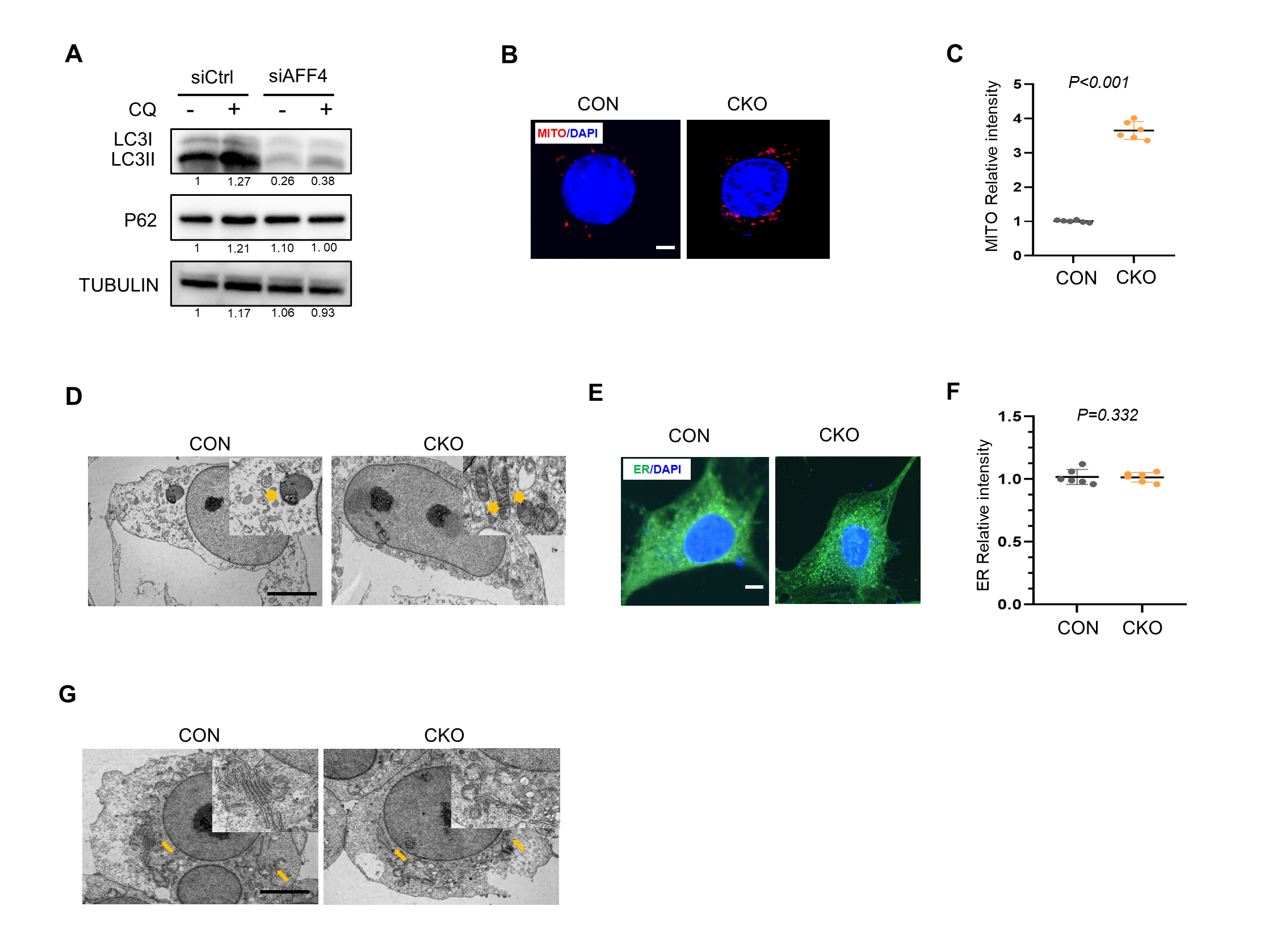

Supplement: S4 Fig — (A) Western blot analysis of autophagy related markers in siCtrl and siAFF4 hMSCs. CQ, chloroquine. (B, C) Representative images and quantification of mitochondria in preadipocytes. n = 6. (D) TEM of preadipocytes from CON and CKO mice, yellow arrow indicating mitochondria. Scale bar, 5 μm. (E, F) Representative images and quantification of endoplasmic reticulum. n = 6. (G) TEM of preadipocytes from CON and CKO mice, yellow arrow indicating endoplasmic reticulum. Scale bar, 5 μm. All quantified data are presented by as mean ± SD. The P values were calculated by two-tailed Student’s t-test. (TIF) [file pgen.1010425.s004.TIF]

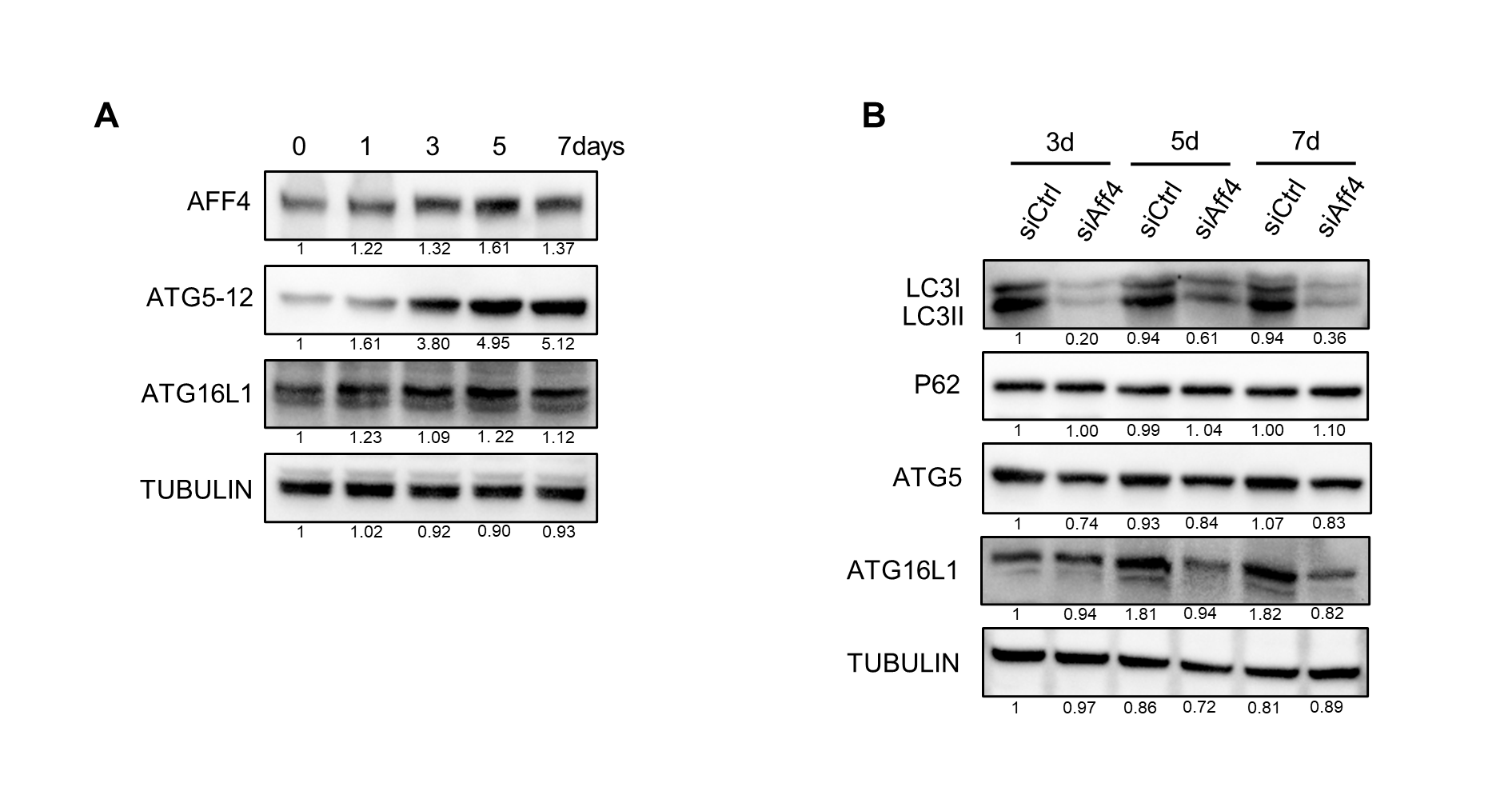

Supplement: S5 Fig — (A) Western blot analysis of AFF4, ATG5 and ATG16L1 expression during adipogenic differentiation of 3T3-L1 cells for 0, 1, 3, 5 and 7 days. (B) Western blot analysis of autophagy related markers in siCtrl and siAff4 3T3-L1 cells after adipogenic differentiation for 3, 5 and 7 days. (TIF) [file pgen.1010425.s005.TIF]

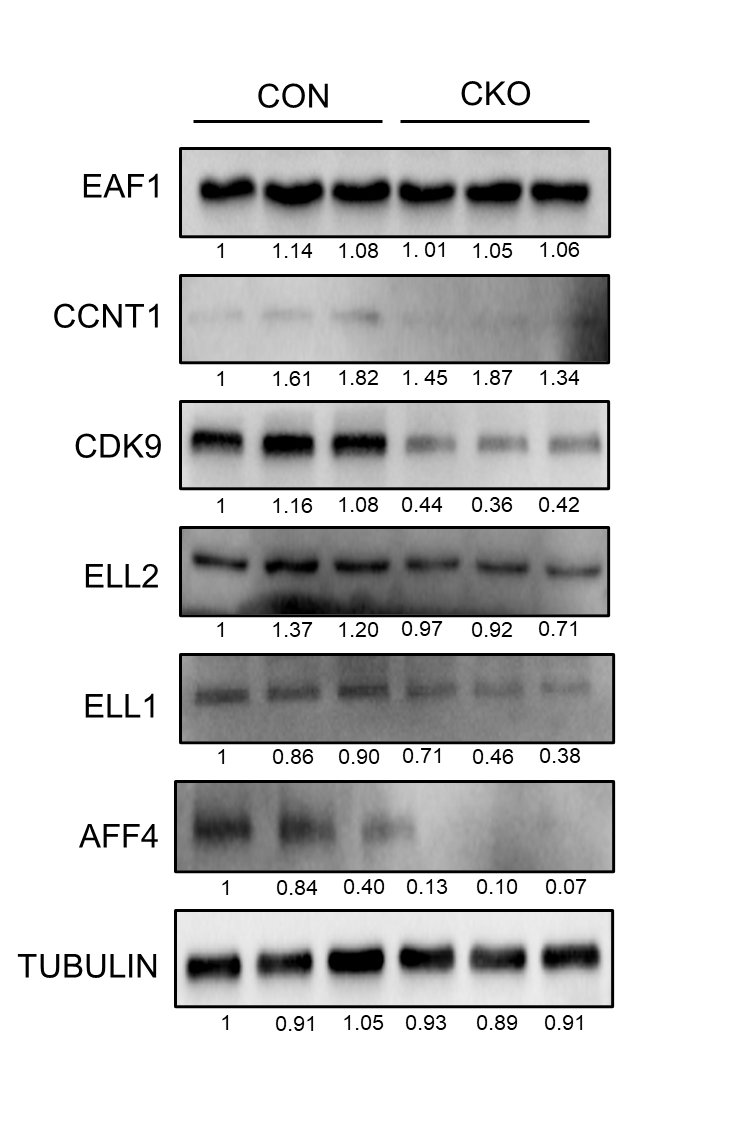

Supplement: S6 Fig — (TIF) [file pgen.1010425.s006.TIF]
