# Supplementary material for: AFF4 regulates cellular adipogenic differentiation via targeting autophagy
Source: PLoS Genet. 2022 Sep 23;18(9):e1010425. doi: 10.1371/journal.pgen.1010425 (PMC9534390; doi:10.1371/journal.pgen.1010425)
Supplement: S1 Table — (DOCX) [file pgen.1010425.s007.docx]

**S1 Table** Primers for RT-qPCR and genotyping.

| **Genes** | **Primers** | **Sequence (5’-3’)** |
| --- | --- | --- |
| *AFF4* | Forward | ACCGTGAAGACCGGAATGTG |
|  | Reverse | GAGAGGAGAGCTAGGTGGGAA |
| *ADIPOQ* | Forward | CCCTCTCTTACAAGCCCATCA |
|  | Reverse | GAGCCAGTCTGGTAGTACATCA |
| *CEBPD* | Forward | CGCGCTGCGGCCAAGTCCTGGTTTTGATTTCACTC |
|  | Reverse | CATGACAGGCCATGGTTAACTACATCAGATACACG |
| *CEBPA* | Forward | TTCACATTGCACAAGGCACT |
|  | Reverse | GAGGGACCGGAGTTATGACA |
| *LPL* | Forward | TCATTCCCGGAGTAGCAGAGT |
|  | Reverse | GGCCACAAGTTTTGGCACC |
| *Aff4* | Forward | ATGAACCGTGAAGACCGGAAT |
|  | Reverse | TGCTAGTGACTTTGTATGGCTCA |
| *Adipoq* | Forward | CGTCACTGTTCCCAATGT |
|  | Reverse | ACCGTGATGTGGTAAGAG |
| *Cebpd* | Forward | GAACCCGCGGCCTTCTA |
|  | Reverse | TGTTGAAGAGGTCGGCGA |
| *Cebpa* | Forward | ACTCCTCCTTTTCCTACCG |
|  | Reverse | AGGAAGCAGGAATCCTCC |
| *Lpl* | Forward | GGTTGCGCGTAGAGAGGATG |
|  | Reverse | CTCACGCTCTGACATGCCTTC |
| *Aff4-flox* | Forward | TGGACATCAAAGAAGCTGTGTTTGGGA |
|  | Reverse | AACCCACCTGTCAGTCCTTCCTCTT |
| *Fabp4WT* | Forward | CAGTGATCATTGCCAGGGAGAACCA |
|  | Reverse | CAAGGCAGCTGCAAGCCCTCTC |
| *Fabp4MUT* | Forward | CAAGGCAGCTGCAAGCCCTCTC |
|  | Reverse | TGGTGCACAGTCAGCAGGTTG |
