# Supplementary material for: AFF4 regulates cellular adipogenic differentiation via targeting autophagy
Source: PLoS Genet. 2022 Sep 23;18(9):e1010425. doi: 10.1371/journal.pgen.1010425 (PMC9534390; doi:10.1371/journal.pgen.1010425)
Supplement: S2 Table — (DOCX) [file pgen.1010425.s008.docx]

**S2 Table** Reagents and resource

| **Reagent or resource** | **Source** | **Identifier** |
| --- | --- | --- |
| **Antibodies** | | |
| α-Tubulin | Beyotime Biotechnology | AF5012 |
| AFF4 | Proteintech | 13197-1-AP |
| AFF4 (For IP) | Bethyl | A302-538A |
| CEBP alpha | Huabio | ET1612-46 |
| Perilipin-1 | Abcam | ab3526 |
| FABP4 | Huabio | ET1703-98 |
| LC3B | Abcam | ab51520 |
| SQSTM1/P62 | Huabio | EM0704 |
| ATG16L1 | Cell Signaling Technology | #8089S |
| ATG5 | Huabio | ET1611-38 |
| KDEL | Huabio | ET7107-86 |
| COX IV | Huabio | ET1701-63 |
| EAF1 | Proteintech | 13787-1-AP |
| CCNT1 | Huabio | HA500313 |
| CDK9 | Huabio | ET1612-78 |
| ELL2 | Proteintech | 12727-1-AP |
| ELL | Proteintech | 51044-1-AP |
| Rabbit IgG | Millipore | CS200581 |
| Goat anti-Rabbit IgG secondary Ab HRP conjugated | Sabbiotech | #L3012 |
| Goat anti-Rabbit Alexa Fluor 550 | Invitrogen | 84541 |
| **Chemicals** | | |
| Chloroquine | Sigma Aldrich | C6628 |
| 3-Methyladenine | MedChemExpress | HY-19312 |
| Fetal bovine serum | Gibco | 10099141 |
| Penicillin-streptomycin | Gibco | 15140163 |
| Minimum Essential Medium α | Cytiva HyClone | SH30265.01 |
| Opti-MEM I | Gibco | 31985062 |
| RNAimax | Invitrogen | 13778075 |
| Hitrans G Polybrene | Gene chem | REVG0001 |
| Puromycin dihydrochloride | Sigma Aldrich | P8833 |
| Dulbecco's Modified Eagle Medium | Cytiva HyClone | SH30243.01 |
| TRIzol reagent | Invitrogen | 15596026 |
| PrimeScript RT reagent Kit with gDNA Eraser | Takara | RR047B |
| Collagenase, Type I | Worthington | LS004196 |
| MitoTracker Red CMXRos | Yeasen | 40741ES50 |
| ER-Tracke Green | Invitrogen | E34251 |
| Insulin | Sigma Aldrich | I9278 |
| IBMX | Sigma Aldrich | [I5879](https://www.sigmaaldrich.cn/CN/zh/product/sigma/i5879) |
| Dexamethasone | Beyotime | ST1258 |
| Matrigel | Corning | 354234 |
| Nile Red | Solarbio | N8440 |
| Oil Red O | Sigma Aldrich | O0625 |
| **Virus strains** |  |  |
| oeAFF4 | Gene chem | N/A |
| oeATG5 | Gene chem | N/A |
| oeATG16L1 | Gene chem | N/A |
| **Small interfering RNA** | | |
| siAFF4 | Santa Cruz | sc-91842 |
| siAff4 | Santa Cruz | sc-140897 |
| siAtg5 | Sangon | N/A |
| siAtg16l1 | Sangon | N/A |
| **Critical commercial assays** | | |
| iTaq Universal SYBR Green Supermix | Bio-rad | 1725124 |
| ACCU-CHEK Aviva blood glucose meter | Roche | N/A |
| Cell Counting Kit-8 | DOJINDO | CK04 |
| Triglyceride assay kit | Nanjing Jiancheng Bioengineering Institute | A110-1-1 |
| EZ-Zyme™ Chromatin Prep Kit | Millipore | #17-375 |
| EZ-Magna ChIP™ HiSens Chromatin Immunoprecipitation Kit | Millipore | #17-10461 |
| TrueSeq mRNA sample preparation kit | Illumina | RS-122-2001 |
| One Step Mouse Genotyping Kit | vazyme | PD101-01 |
| **Experimental models: Organisms/Strains** | | |
| Mouse: C57BL/6 Aff4^flox/+^ | Biocytogen | N/A |
| Mouse: C57BL/6 Fabp4-Cre | The Jackson Laboratory | 018965 |
| Nude mouse: BALB/c | CHENGDU DOSSY EXPERIMENTAL ANIMALS | N/A |
| **Experimental models: Cell lines** | | |
| Human MSCs | Cyagen | HUXMA-01001 |
| 3T3-L1 cells | ATCC | CL-173 |
| **Software and algorithms** |  |  |
| Image J | NIH | https://imagej.nih.gov/ij/ |
| GraphPad Prism 8 | GraphPad Software | https://www.graphpad.com/ |
| SPSS Statistics | IBM | https://www.ibm.com/products/spss-statistics |
| GSEA 4.1 | Broad Institute | http://www.broad.mit.edu/GSEA |
| IGV 2.12.3 | Broad Institute | https://software.broadinstitute.org/software/igv/ |
| R 4.0 | N/A | https://www.r-project.org/ |
| **Deposited data** |  |  |
| RNA seq | NCBI’s Gene Expression Omnibus | GSE197354 |
| ChIP seq | NCBI’s Gene Expression Omnibus | GSM1527701, GSM1961563, GSM1961564 |
